# A Site-Specific, Single-Copy Transgenesis Strategy to Identify 5′ Regulatory Sequences of the Mouse Testis-Determining Gene *Sry*

**DOI:** 10.1371/journal.pone.0094813

**Published:** 2014-04-17

**Authors:** Alexander Quinn, Kenichi Kashimada, Tara-Lynne Davidson, Ee Ting Ng, Kallayanee Chawengsaksophak, Josephine Bowles, Peter Koopman

**Affiliations:** 1 Institute for Molecular Bioscience, The University of Queensland, Brisbane, Australia; 2 Department of Pediatrics and Developmental Biology, Tokyo Medical and Dental University, Bunkyo-ku, Tokyo, Japan; Leibniz Institute for Age Research - Fritz Lipmann Institute (FLI), Germany

## Abstract

The Y-chromosomal gene *SRY* acts as the primary trigger for male sex determination in mammalian embryos. Correct regulation of *SRY* is critical: aberrant timing or level of *Sry* expression is known to disrupt testis development in mice and we hypothesize that mutations that affect regulation of human *SRY* may account for some of the many cases of XY gonadal dysgenesis that currently remain unexplained. However, the *cis-*sequences involved in regulation of *Sry* have not been identified, precluding a test of this hypothesis. Here, we used a transgenic mouse approach aimed at identifying mouse *Sry* 5′ flanking regulatory sequences within 8 kb of the *Sry* transcription start site (TSS). To avoid problems associated with conventional pronuclear injection of transgenes, we used a published strategy designed to yield single-copy transgene integration at a defined, transcriptionally open, autosomal locus, *Col1a1*. None of the *Sry* transgenes tested was expressed at levels compatible with activation of *Sox9* or XX sex reversal. Our findings indicate either that the *Col1a1* locus does not provide an appropriate context for the correct expression of *Sry* transgenes, or that the *cis-*sequences required for *Sry* expression in the developing gonads lie beyond 8 kb 5′ of the TSS.

## Introduction

The Y-chromosomal testis-determining gene *SRY/Sry* has a unique importance in mammalian biology, providing the key to sexual reproduction that in turn acts as the engine for genetic recombination and natural selection. *Sry* provides the pivot point for sex development in XY mammalian embryos by stimulating the differentiation of testes rather than ovaries from the embryonic gonadal anlagen, the genital ridges [1]. Once testes have formed, differentiation of the embryo as a male is promoted by hormones produced by the Leydig cells. In embryos where *Sry* is absent or non-functional the genital ridges develop as ovaries and female differentiation follows [2], [3].

More than 20 years have elapsed since *SRY/Sry* was discovered [4], [5], but relatively little is understood at the molecular or cellular levels regarding how it achieves its important role. The critical function of *Sry* is to up-regulate transcription of *Sox9* in a subset of genital ridge cells [6], [7]. Once *Sox9* is activated, the transcription factor it encodes appears to co-ordinate the sequence of molecular events leading to Sertoli cell differentiation [8], [9]. *Sry* can therefore be viewed as a switch whose activity is required only briefly, after which its work is done.

Recent studies have shed some light on the mechanism by which SRY activates *Sox9* transcription. SRY protein contains an HMG DNA-binding domain that binds to regulatory elements upstream of *Sox9* in conjunction with the partner factor NR5A1 (SF1) [7]. SRY activity also evidently rests on a transcriptional trans-activation domain at its C-terminus [10], [11]. Once *Sox9* transcription is activated, it is sustained through a positive-feedback loop involving SOX9 protein (also an HMG transcription factor), NR5A1 and the same regulatory elements upstream of *Sox9*.

It has become apparent that *Sry* expression needs to be regulated precisely in order to activate *Sox9* transcription and initiate the testis-determining pathway, rather than allowing the ovarian-determining pathway to take hold. In the mouse, *Sry* expression starts at 10.5 dpc in somatic cells of XY genital ridges, reaches a peak at 11.5 dpc and wanes by 12.5 dpc [12]–[16]. If *Sry* expression is delayed by as little as a few hours either experimentally [17] or through breeding of Y chromosomes from certain strains onto a C57BL/6 genetic background [18], [19], testis differentiation is compromised or blocked completely in favour of ovarian development. The current working model is that SRY expression in each cell must reach a threshold level within a defined window of time in order for *Sox9* to be activated cell-autonomously [20]. Based on these findings it seems likely that mutations affecting human *SRY* expression may account for some of the many cases of XY gonadal dysgenesis that currently remain unexplained.

A handful of transcription factors have been implicated in regulating *Sry* expression, including WT1 [21], NR5A1 [7], GATA4/FOG2 [22], and SIX1/SIX4 [23]. In addition, insulin growth factor signalling [24], mitogen-activated protein kinase signalling [25] in conjunction with the associated stress response protein GADD45γ [26], [27], and chromatin remodelling factors such as histone demethylase JMJD1A [28] and polycomb protein CBX2 [29], have all been implicated in *Sry* regulation. However, the critical transcription factors and their target binding *cis*-regulatory sites have not been identified, clouding our understanding of how *Sry* is regulated. Further, *Sry* resides on the Y chromosome in a quicksand of genetic drift, rendering conventional bioinformatic tools used for identifying important DNA sequence motifs by cross-species comparison largely useless.

Here, we use a transgenic mouse approach aimed at identifying mouse *Sry* 5′ flanking regulatory sequences. In previous studies, a 14 kb genomic DNA fragment including *Sry* was shown to cause sex reversal in XX transgenic mice [1], [6], [11]. In addition, reporter transgenes lacking the *Sry* coding region but including the proximal 8 kb of the *Sry* 5′ region have been shown to be expressed with a similar spatial and temporal pattern to endogenous *Sry* [6], [30]. Based on these studies, our starting assumption was that all *cis-*sequences required for correct expression of *Sry* lie within 8 kb 5′ of the transcription start site (TSS). The strategy used here is based on *Sry* genomic DNA fragments that differ in their 5′ flanking sequence content, and assaying the ability of these fragments to direct male sex development in XX embryos.

Previous studies also found variation in the ability of the 14 kb *Sry* transgene to induce XX sex reversal [1], [6], [11], most likely because in those studies it was not possible to create mice with a predetermined number of copies of the transgene, nor ensure a consistent site of transgene integration. These technical difficulties, in combination with the restricted and transient expression of *Sry*, have complicated the conduct and interpretation of experiments designed to locate *Sry* regulatory sequences.

To avoid these problems, we adopted a published strategy designed to yield single-copy transgene integration at a defined autosomal locus, *Col1a1* [31]. However, we found that none of the *Sry* transgenes tested was expressed at levels compatible with XX sex reversal. Our experiments suggest either that the *Col1a1* locus, although supposedly in an open transcriptional state, is not an appropriate context for the correct epigenetic and/or transcriptional activation of *Sry* transgenes, or that the assumption that the *cis-*sequences required for *Sry* transcription in the genital ridges lie within 8 kb 5′ of the TSS is incorrect.

## Materials and Methods

### Ethics statement

All procedures involving animals and their care conformed to institutional, state and national guidelines. This study was approved by the University of Queensland Animal Ethics Committee (Permit Numbers: IMB/076/11/ARC/NHMRC, IMB/434/09/ARC/QSG/UQ/BREED (NF) and IMB/087/10/NHRMC/ARC (NF)).

### ES cells

**Figure 1:**
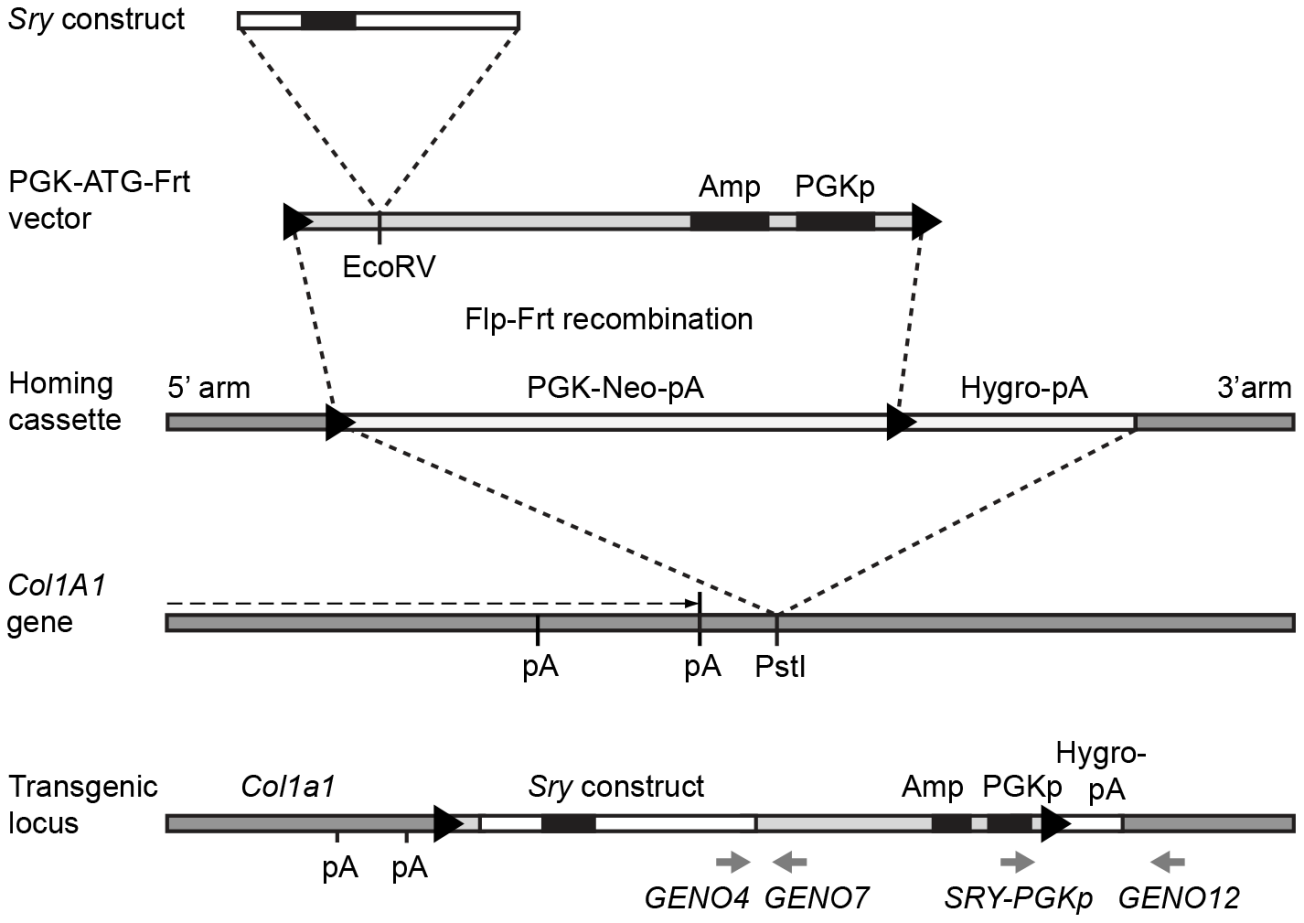
Site-specific integration of *Sry* transgenes 3′ to the *Col1A1* gene. KH2 ES cells carry an Frt-PGK-neo-pA-Frt-hygro-pA “homing” cassette at a *Pst*I site downstream of the *Col1A1* locus. *Sry* transgene constructs were cloned into a PGK-ATG-Frt vector, and then this was integrated into the homing site within the KH2 cells by co-electroporation with a FlpE transient expression vector (pCAGGS-FlpE-puro). Flp-Frt homologous recombination resulted in loss of the PGK-neo-pA cassette and insertion of the *Sry*-PGK-ATG-Frt vector bearing the transgene into the *Col1A1* locus in the ES cells. Black arrowheads indicate Frt sites, black boxes indicate exons, grey arrows and italics indicate primer sites for genotyping of ES cells and mice. Amp, ampicillin resistance. Neo, neomycin resistance. Hygro, hygromycin resistance. pA, polyadenylation signal. PGKp, PGK promoter. Not drawn to scale.

An ES cell line (KH2) with an Frt-PGK-neo-pA-Frt-hygro-pA transgene homing cassette integrated ∼0.5 kb downstream of the 3′ untranslated region (UTR) of the *Col1a1* gene was kindly provided by R. Jaenisch and K. Hochedlinger (Fig. 1) (#MES4304, Thermo Scientific Open Biosystems) [31]. KH2 is an F1 ES cell line derived from a C57BL/6 and 129svJae cross. The cassette had been inserted by homologous recombination, with correctly targeted cells selected on the basis of neomycin resistance [31]. In conjunction with a Flp recombinase, this cassette allows for site-specific Flp-Frt recombination with a targeting vector consisting of an Frt site, a gene of interest, and a PGK promoter and ATG initiation codon that enable transcription (and selection for) the hygromycin resistance gene when correctly integrated. KH2 cells were grown on hygromycin-resistant MEFs at 37°C (5% CO_2_) in high-glucose DMEM media (GIBCO) supplemented with 20% fetal calf serum, 1% penicillin/streptomycin, 1% β-mercaptoethanol, 1% GlutaMax, 1% non-essential amino acids and 1% sodium pyruvate.

### Sry-PGK-ATG-Frt constructs

**Figure 2:**
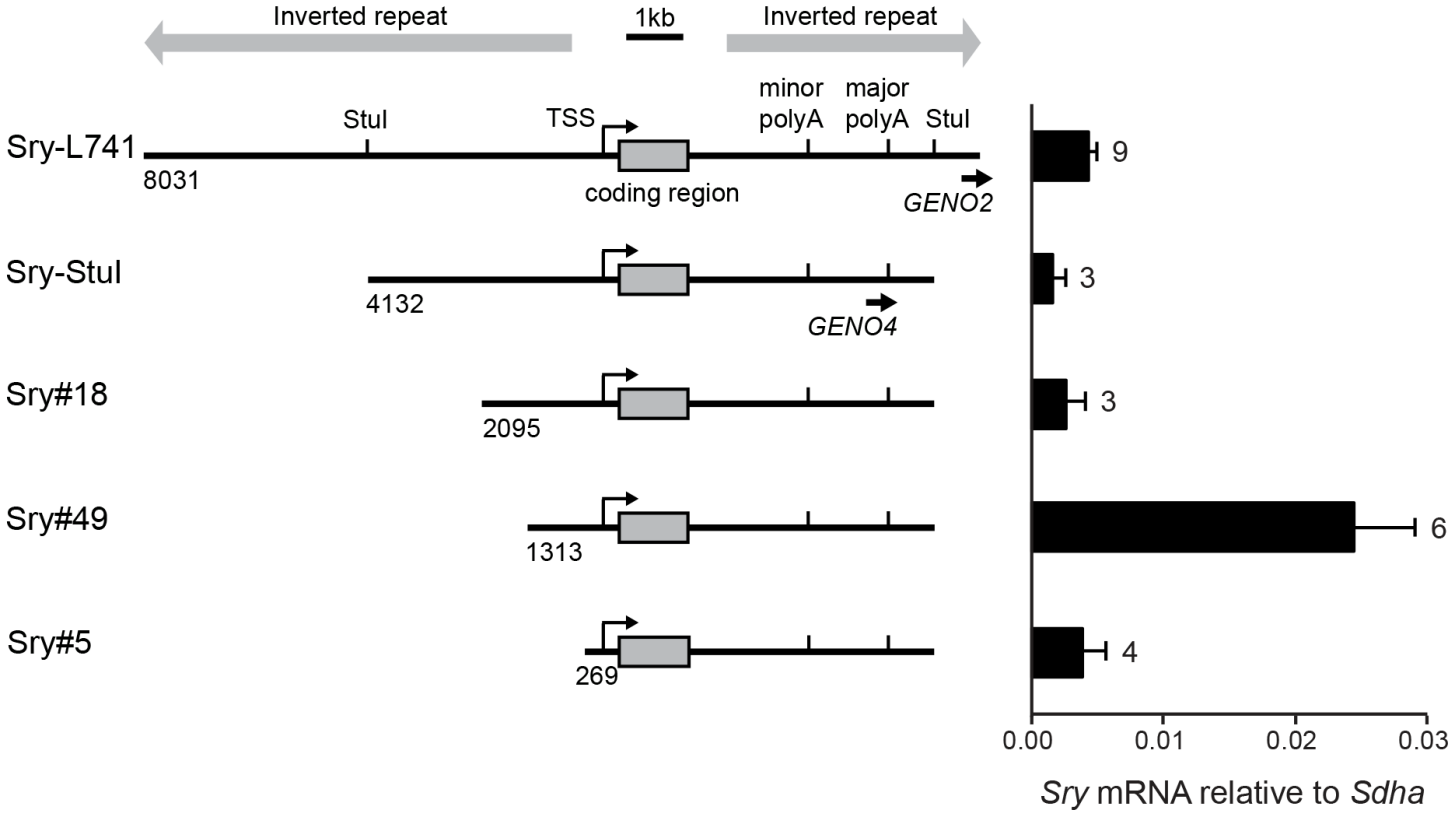
Transgene constructs and relative transcription of *Sry* in the five transgenic mouse lines. Left panel shows the structure of the five transgene constructs integrated into the *Col1a1* locus, featuring sequential deletions of the *Sry* 5′ region. Length of sequence 5′ to the transcriptional start site (TSS) is indicated beneath the constructs (base-pairs). Grey box, *Sry* coding region. PolyA, polyadenylation signal. Black arrows and italics indicate primer sites used for genotyping. Grey arrows indicate inverted repeat sequences flanking the *Sry* coding region. Right hand panel shows corresponding qRT-PCR analysis of *Sry* mRNA levels in samples (gonad and mesonephros pairs) collected at 11.5 dpc (2^−**Δ**Ct^, normalized against *Sdha*). Error bars are 95% confidence intervals. Numbers beside bars indicate sample size (individual gonad-mesonephros pairs).

DNA fragments containing the *Sry* gene and flanking sequences were prepared from the 14.6 kb genomic clone L741 (NCBI: X67204) [5]. Five *Sry* fragments with 5′ regions of varying length were prepared (Fig. 2). Sry-StuI was excised from L741 by *Stu*I digestion. Because the sequences flanking the *Sry* locus are palindromic, three fragments with progressively shorter 5′ arms (Sry#18, Sry#49, Sry#5) were prepared by 5′ exonuclease digestion of the Sry-StuI fragment. All five fragments were blunted by 3′ exonuclease digestion and cloned into the *EcoR*V site of a PGK-ATG-Frt targeting vector (#MES4490, Thermo Scientific Open Biosystems). Direction of the fragments was confirmed by direct sequencing using primers MES4490-F and MES4490-R on either side of the *EcoR*V site in the vector. Direction of the Sry-StuI insert was confirmed by *Bgl*II digestion because of the palindromic sequence of the *Sry* 5′ and 3′ flanking regions. Integrity of the 1188 bp *Sry* protein-coding sequence was verified for all cloned fragments by direct sequencing using primers SRY-CDS-F and SRY-CDS–R. All primers used for genotyping and sequencing are presented in Table 1, and the location of some primer sites are indicated in Figures 1 and 2.

**Table 1:** Genotyping and sequencing primer information.

Primer	Sequence (5′-3′)	Primer site
MES4490-F	CGCGCAATTAACCCTCACTA	PGK-ATG-Frt vector, 5′ to EcoRV insertion site
MES4490-R	GGCTTCAATGCCTGCTTGTTAC	PGK-ATG-Frt vector, 3′ to EcoRV insertion site
SRY-PGK	CCCGGCATTCTGCACGCTTC	PGK promoter of PGK-ATG-Frt vector
COL1A1-GENO7	CCCAGCTTCACCAGTTCAAT	Col1a1 locus, 5′ to integrated homing cassette
COL1A1-GENO12	CTACCCCTCCATGTGTGACC	Col1a1 locus, 3′ to integrated homing cassette
ROSA26-1	AAAGTCGCTCTGAGTTGTTAT	Rosa26 locus (intron 1)
ROSA26-3	GGAGCGGGAGAAATGGATATG	Rosa26 locus (intron 1)
SRY-GENO2	GGGAGGGGTTAGTGGTTAGTG	3′ end of Sry-StuI, #18, #49, #5 fragments
SRY-GENO4	GTTAGCTGTCCAAGGTTCAGG	3′ end of Sry-L741 fragment
SRY-GENO7	CTTTATGCTTCCGGCTCGTATG	PGK-ATG-Frt vector, 3′ to EcoRV insertion site
SRY-CDS-F	GCCAGATCTAAGAGACAAGTTTTGGGACTGGTGACA	Flanking 5′ end of Sry protein coding sequence
SRY-CDS-R	AGGGGGAGTGTTGGCATAGGTAGGAGA	Flanking 3′ end of Sry protein coding sequence
SX-F	GATGATTTGAGTGGAAATGTGAGGTA	Sly locus (intron 8), Xlr locus (intron 6)
SX-R	CTTATGTTTATAGGCATGCACCATGTA	Sly locus (intron 8), Xlr locus (intron 6)

### Site-specific recombination of Sry constructs into ES cells

Approximately 0.5–1×10^7^ KH2 cells were electroporated with 50 µg Sry-PGK-ATG-Frt plasmid and 25 µg FlpE recombinase-expressing vector (pCAGGS-FlpE-puro, #MES4488, Thermo Scientific Open Biosystems) at 500 V and 25 µF using two pulses in a Gene Pulser Xcell Electroporation System (Bio-Rad). After 48 h growth, cells were treated with 140 µg/ml hygromycin to select for cells in which the targeting vector had been inserted by Flp-Frt recombination. Integration of Sry-PGK-ATG-Frt constructs into KH2 cells was identified by PCR amplification of a 1.5 kb product with primers SRY-PGK and COL1A1-GENO12.

### Generation of chimeras and transgenic lines

Hygromycin-selected ES cells were injected into wild type C57Bl/6J donor blastocysts (10–15 ESC/blastocyst), and blastocysts were transferred into a 0.5 or 2.5 dpc pseudopregnant CD1 female. Chimeric offspring were identified by coat colour then bred to C57Bl/6 females to generate the first generation; pups in which germ line transmission had occurred were identified by genotyping for presence of the transgene. Heterozygous mice were used to establish lines for analysis.

### Genotyping of transgenic offspring

Integration of the *Sry* transgene in offspring was determined by PCR amplification of a 261 bp product with primers SRY-GENO4 and SRY-GENO7 (for the Sry-L741 line) or a 201 bp product with primers SRY-GENO2 and SRY-GENO7 (for all other lines), using genomic DNA extracted from ear notch tissue of juveniles. Sex of juveniles was determined by examination of external reproductive anatomy. Transgenic studs were occasionally mated with transgenic females of the same line. Transgenic offspring homozygous for the *Sry* transgene were distinguished from heterozygotes using primers COL1A1-GENO7 and COL1A1-GENO12, which span the insertion site of the *Sry-*PGK-ATG-Frt construct, 3′ to the *Col1a1* gene. These primers amplify a 329 bp product from the wild type allele, but do not amplify a product from *Col1a1* alleles containing the inserted *Sry-*PGK-ATG-Frt construct (which is several kilobases in length). Thus, the 329 bp product was amplified in heterozygous transgenics but not homozygotes. A second primer pair, ROSA26-1 and ROSA26-3 [32], was included in the genotyping PCRs, to amplify a 603 bp product from the *Rosa26* locus. This larger product served as a positive control for successful amplification, reducing the likelihood of incorrect genotype calls in the event of PCR failure. For each transgenic line, the entire *Sry* coding region was amplified, cloned and sequenced from a representative transgenic XX embryo to again verify the integrity of the protein-coding sequence.

### Timed matings and dissection

Embryos were collected from timed matings of transgenic studs with outbred CD1 strain females, with noon of the day on which the mating plug was observed designated as 0.5 days *post coitum* (dpc). For more accurate staging, the tail somite (ts) stage of the embryo was determined by counting the number of somites posterior to the hind limb. Using this method, 10.5 dpc is approximately equivalent to 8 ts, 11.5 dpc to 18 ts, and 12.5 dpc to 30 ts [13]. For all embryos, chromosomal sex was determined by PCR genotyping with primers SX-F and SX-R [33], using genomic DNA extracted from tail tissue. From 12.5 dpc onwards, embryos were also sexed by morphological assessment of the gonads. Gonad pairs were dissected and immediately stored in RNAlater RNA stabilization solution (Invitrogen) until required for gene expression analysis by quantitative RT-PCR. For embryos at or prior to 12.5 dpc, gonads were collected with mesonephros attached, whereas gonadal tissue only was collected from embryos beyond this stage.

### RNA extraction, cDNA synthesis, and quantitative real-time RT-PCR

Gonadal tissue from each embryo was processed and analyzed individually. Total RNA was extracted and subjected to DNase treatment using an RNeasy Micro kit (Qiagen) in accordance with the manufacturer's instructions, and quantified with a NanoDrop spectrophotometer (NanoDrop Technologies). cDNA was synthesized by reverse transcription with SuperScript III and random hexamers (Invitrogen), from 100 ng total RNA (for analyses of precisely-staged embryos between 8–30 ts, within a single transgenic line) or from 300 ng total RNA (for all other analyses, at 11.5 or 13.5 dpc). All qRT-PCR reactions were performed in triplicate using SYBR Green PCR master mix (Invitrogen) and 150 nM each of forward and reverse primers, and analyzed on a Viia7™ Real-Time PCR System (Invitrogen). Sry cDNA was amplified with primers 5′-TTATGGTGTGGTCCCGTGGT and 5′-GGCCTTTTTTCGGCTTCTGT [34] and Sox9 cDNA was amplified with primers 5′-AGTACCCGCATCTGCACAAC and 5′-TACTTGTAATCGGGGTGGTCT. Relative cDNA levels were determined by calculating 2−ΔCt values relative to the house-keeping gene Sdha, using primers 5′-TGTTCAGTTCCACCCCACA and 5′-TCTCCACGACACCCTTCTGT. Sdha was verified previously as a suitable normalization gene for gonadal tissue [35]. qRT-PCR data is presented as the mean 2−ΔCt value for multiple individual embryos (minimum of 3). Sample sizes are indicated in relevant figures.

### Section immunofluorescence

Whole embryos were collected at 11.5 dpc and fixed in 4% paraformaldehyde in phosphate-buffered saline (PBS) at 4°C overnight, washed three times with PBS at 4°C, then dehydrated and embedded in paraffin. Section immunofluorescence was performed on 7 µm-thick sagittal sections as described previously [16]. Rabbit anti-SRY (1∶100) [16] and goat anti-rabbit Alexa Fluor 488 (1∶200, Invitrogen) were used as primary and secondary antibodies, respectively. Images were captured using a BX51 fluorescence microscope fitted with a DP70 camera (Olympus), and compiled using the associated software (Olympus) and Adobe Photoshop CS6 version 16.0 (Adobe) software.

## Results

We created five transgenic constructs containing the mouse *Sry* coding region (1.2 kb) with sequential deletions of the 5′ flanking sequence; Sry-L741 (8.0 kb of 5′ sequence), Sry-StuI (4.1 kb), Sry#18 (2.1 kb), Sry#49 (1.3 kb), and Sry#5 (0.3 kb) (Fig. 2). All constructs also included 3′ flanking sequences for *Sry*, encompassing both the minor and major polyadenylation sites [13], [14] (L741, 5.2 kb of 3′ sequence; all others, 4.2 kb). Constructs were integrated in single copy at the same autosomal locus (3′ to the *Col1a1* gene) by homologous recombination in ES cells bearing a homing cassette at that location [31]. We established transgenic mouse lines from blastocysts injected with the ES cells, then analysed the lines for XX sex reversal and for level and timing of *Sry* transgene expression, in an attempt to identify *cis-*sequences required for correct regulation of *Sry.*

### Genotype and phenotype analysis of transgenic offspring

Breeding lines for each of the five *Sry* transgenes were maintained by mating of transgenic males with wild type CD1 females. To identify any instances of XX sex reversal, a total of 495 juvenile offspring of these matings (*n* = 82 for Sry-L741, *n* = 81 for Sry-StuI, *n* = 123 for Sry#18, *n* = 131 for Sry#49, *n* = 78 for Sry#5) were sexed and genotyped to determine both chromosomal sex (XX or XY) and presence or absence of an *Sry* transgene (Table 2). The ratios of transgenic to wild type (251∶244) and XX to XY (249∶246) offspring were both very close to 1∶1. None of 113 XX Sry-transgenic offspring developed as male, indicating that all five variants of the *Sry* transgenes in single-copy at the *Col1a1* locus were unable to induce XX sex reversal. Moreover, no indications of sex reversal were observed in the gross structure of dissected gonads from 13.5 dpc XX embryos transgenic for Sry-L741, Sry#18 or Sry#49.

**Table 2:** Numbers of transgenic and wild type animals bred from the five *Sry* transgenic lines.

Transgene	5′ length (kb)	Transgenic		Wild type	
		XY	XX	XY	XX
Sry-L741	8.0	22	21	19	20
Sry-StuI	4.1	19	17	24	21
Sry#18	2.1	42	25	25	31
Sry#49	1.3	31	32	28	40
Sry#5	0.3	24	18	12	24
Totals		138	113	108	136

To test whether increased dosage of Sry-L741 or Sry#49 were able to induce XX sex reversal, transgenic males were mated with transgenic females of the same line to generate XX offspring homozygous for the transgene (at an expected proportion of 0.125 XX offspring/litter). Three of 19 embryos examined at 13.5 dpc were determined by genotyping as XX and homozygous for Sry-L741, and four of 30 embryos examined at 13.5 dpc were XX and homozygous for Sry#49. No indications of female to male sex reversal were observed in the gross gonadal morphology of the XX transgenic homozygotes for either line.

### Transgene expression analysis for gonads at 11.5 dpc

**Figure 3:**
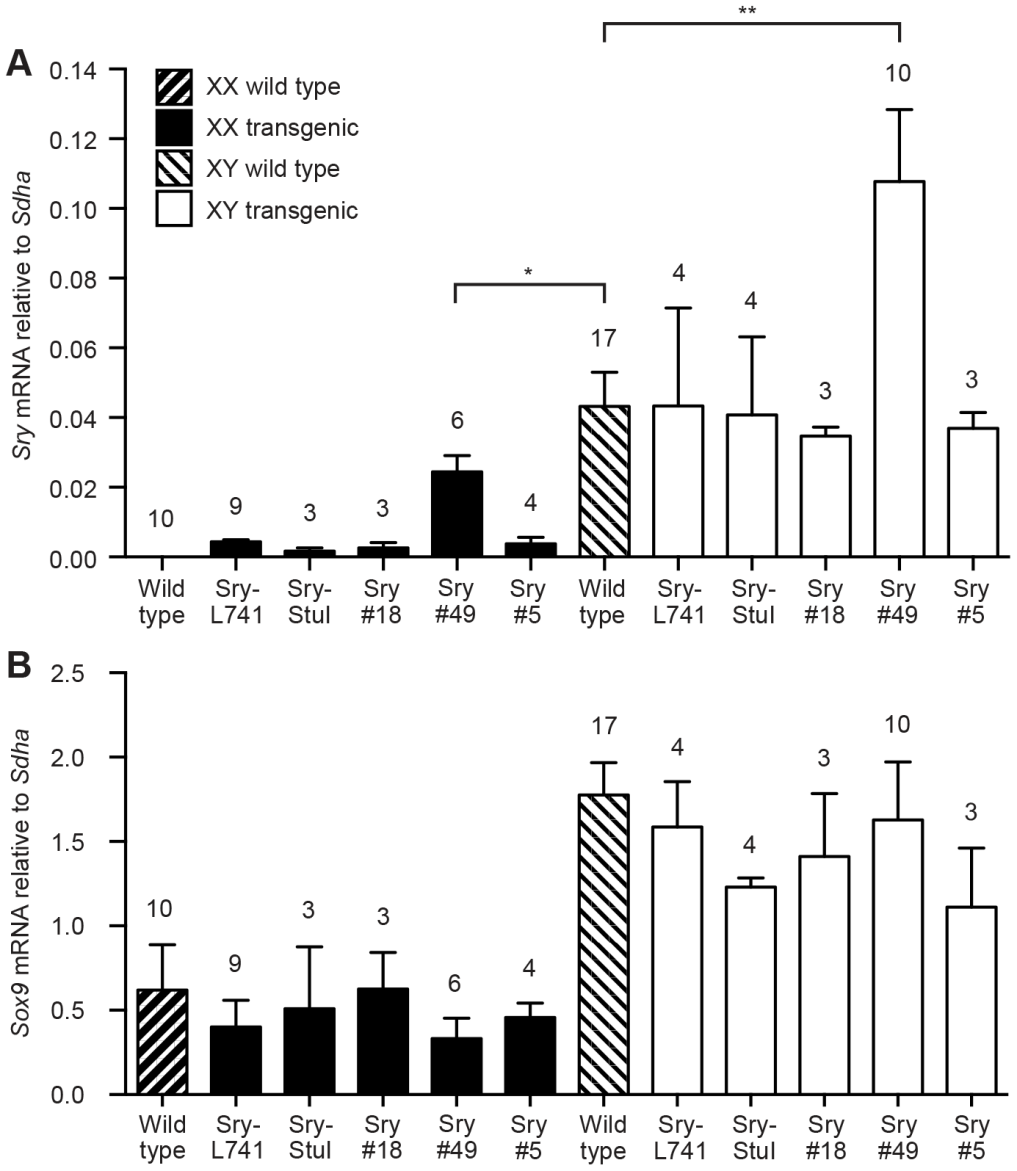
qRT-PCR analysis of *Sry* and *Sox9* expression at 11.5 dpc. (A) Total *Sry* mRNA expression for wild type and transgenic XX (black) and XY (white) embryos. (B) *Sox9* mRNA expression for the same embryos. Relative expression is measured as 2^−**Δ**Ct^ values, normalized against *Sdha*. Numbers above bars indicate sample size (individual gonad-mesonephros pairs). Error bars are 95% confidence intervals. *P<0.01, **P<0.001, Student's unpaired t-test.

For all five transgenic lines, gonadal expression of *Sry* and *Sox9* was analysed by qRT-PCR at 11.5 dpc, the time point when endogenous *Sry* expression reaches its maximum in wild type XY embryos. In XX gonads, the Sry#49 transgene showed markedly higher *Sry* mRNA expression than the four other transgenes, all of which showed very low expression (Fig. 2). Although Sry#49 was the most strongly expressed of the five transgene constructs in XX gonads, the total level of *Sry* mRNA was approximately half that found in wild type XY gonads at 11.5 dpc (Fig. 3A). Analysis of total *Sry* mRNA levels in XY gonads, comprising both endogenous *Sry* and transgenic *Sry* mRNA, similarly revealed that Sry#49 produced the highest expression for the five transgenic lines (Fig. 3A). The total *Sry* mRNA level in XY Sry#49 gonads was strikingly higher than for XY wild type gonads, and presumably reflected the combined effect of endogenous *Sry* mRNA and Sry#49 mRNA. Total *Sry* mRNA levels for XY Sry-L741, Sry-StuI, Sry#18 and Sry#5 gonads did not differ significantly from wild type XY levels.

There was no apparent effect of *Sry* transgene expression on the level of *Sox9* transcription in the XX transgenic gonads at 11.5 dpc, even for Sry#49, the line exhibiting the highest transgene expression (Fig. 3B). For all XX transgenic lines, *Sox9* mRNA expression did not differ significantly from the basal level of *Sox9* expression in XX wild type gonads, and was far less than for XY gonads. Similarly, expression of the *Sry* transgenes in XY gonads did not cause any further increase in *Sox9* expression above the level for XY wild type gonads at 11.5 dpc. The failure of the *Sry* transgenes to upregulate *Sox9* in the gonads evidently underlies the lack of XX sex reversal in the transgenic lines.

Given the lack of *Sox9* upregulation and lack of XX sex reversal observed for all five transgenic lines, subsequent molecular analyses were restricted to Sry-L741, the transgene bearing the longest *Sry* 5′ region sequence, and Sry#49, the transgene producing the highest level of *Sry* transcription.

### Sry protein expression analysis of transgenic gonads

**Figure 4:**
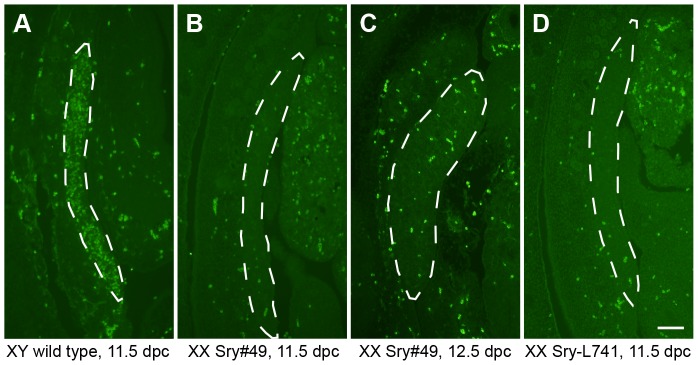
Expression of SRY protein in transgenic mouse gonads. Expression was visualised by immunofluorescent staining of sagittal embryo sections. Dashed outline indicates gonad tissue. (A) XY wild type embryo at 11.5 dpc. (B) XX Sry#49 embryo at 11.5 dpc. (C) XX Sry#49 embryo at 12.5 dpc. (D) XX Sry-L741 embryo at 11.5 dpc. Scale bar, 100 µm.

Immunofluorescent antibody staining of sectioned embryos failed to detect Sry protein expression in XX Sry-L741 gonads at 11.5 dpc, or in XX Sry#49 gonads at 11.5 and 12.5 dpc, but did identify Sry expression along the entire length of a control XY wild type gonad at 11.5 dpc (Fig. 4). This result is consistent with the very low mRNA levels for XX transgenic gonads revealed by qRT-PCR, and suggests the level of Sry protein per cell, even for XX Sry#49 gonads, was probably too low to be detectable by immunofluorescence. The sequence of the transgenic *Sry* coding region in each of the transgenic lines was also verified as being intact, eliminating the possibility that introduced mutations had caused protein instability and/or loss of function. It is possible that Sry protein was not expressed at all, but based on the apparently very low mRNA expression per cell, we consider it likely that Sry protein levels were simply below a threshold for detection by immunofluorescence.

### Temporal transgene expression analysis

**Figure 5:**
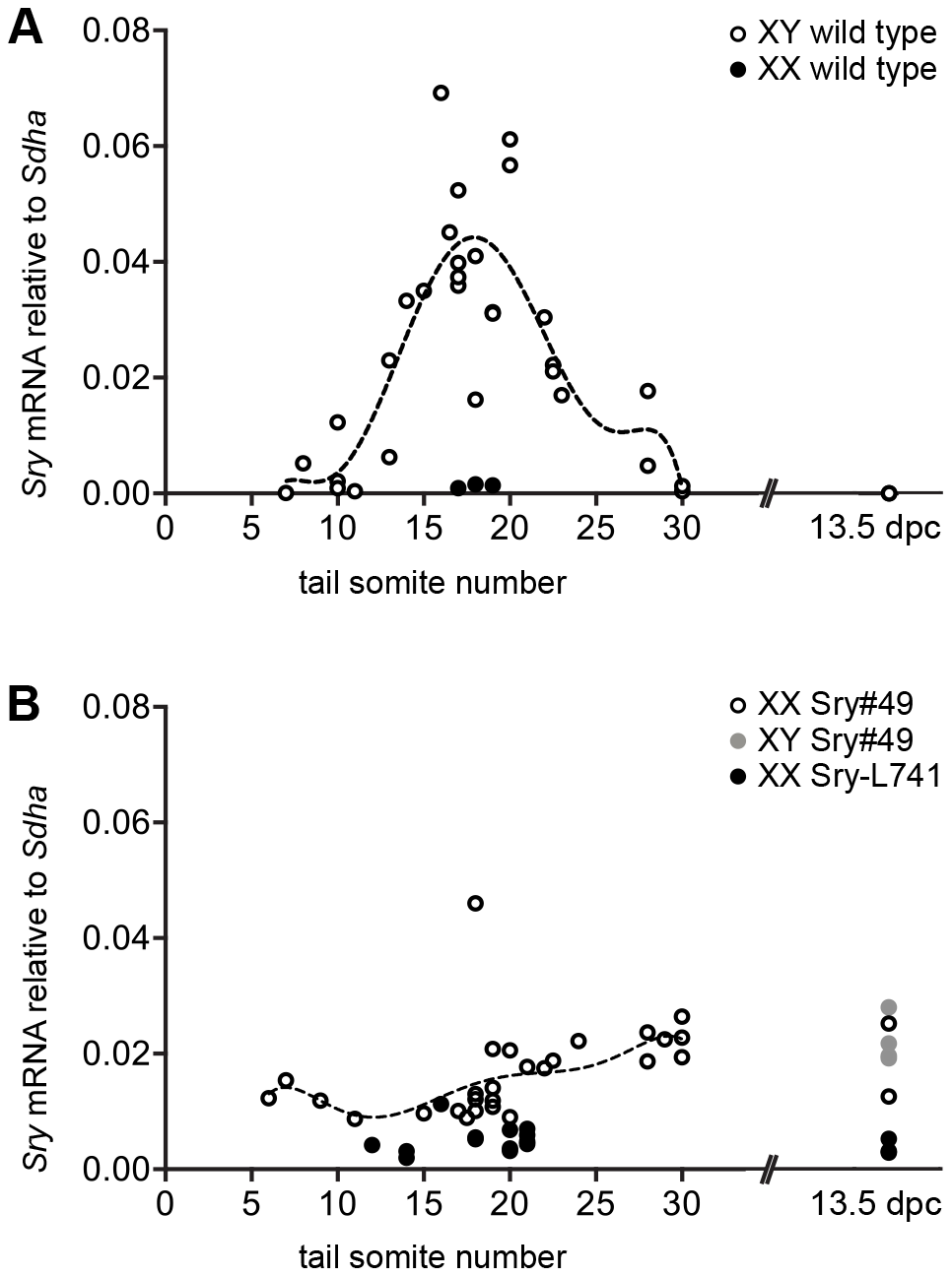
Temporal analysis of gonadal *Sry* expression. qRT-PCR (2^−**Δ**Ct^, normalized against *Sdha*) was performed for gonad-mesonephros pairs from precisely staged embryos between 10.5 dpc (∼8 ts) and 12.5 dpc (∼30 ts), and for gonad pairs at 13.5 dpc. Each circle represents a single sample. (A) Wild type XY (white) and XX (black) gonads. Dashed line shows curve of fit generated by non-linear regression for XY wild type gonads (5–30 ts, 6^th^ order polynomial function, least squares fit, *R^2^* = 0.7012). (B) XX Sry#49 (white), XY Sry#49 (grey), and XX Sry-L741 (black) gonads. XY Sry#49 gonads are included at 13.5 dpc only, as endogenous *Sry* expression would mask transgenic *Sry* expression at the earlier time points. Dashed line shows curve of fit generated by non-linear regression for XX Sry#49 gonads (5–30 ts, 6^th^ order polynomial function, least squares fit, *R^2^* = 0.2081).

None of the five transgenes were evidently transcribed at a level sufficient to upregulate *Sox9* expression and effect XX sex reversal, but a further question remained over the ability of the transgenes to emulate the temporal expression pattern of endogenous *Sry*, which is switched on at approximately 10.5 dpc, peaks at 11.5 dpc, and ceases at 12.5 dpc (Fig. 5A). To test this, we compared transgene expression in XX gonads with *Sry* expression in XY wild type gonads from precisely staged embryos between 10.5 and 12.5 dpc, using wild type/transgenic littermates whenever possible. Sry#49 was examined most thoroughly because it produced the highest level of *Sry* mRNA expression of the five transgenes, enabling the temporal pattern of expression to be established clearly (Fig. 5B). A smaller sample of precisely staged Sry-L741 gonads was also analysed, since this transgene bears the longest *Sry* promoter sequence of the variants. A few gonads from the later stage of 13.5 dpc were included in this analysis for both the Sry#49 and Sry-L741 lines.

Sry#49 displayed roughly equivalent expression in gonads from the earliest stages examined (6–7 ts) right through to the latest stage (13.5 dpc) - that is, prior to and well after the window of endogenous *Sry* expression - and did not peak at 11.5 dpc. A single gonad pair at 18 ts gave a strikingly higher expression value for Sry#49, but this sample appeared to be an outlier amongst many other gonads analysed at 17–19 ts. If any pattern could be discerned for Sry#49, it was possibly a subtle increase in gonad expression as time progressed. The few Sry-L741 gonads analysed produced much lower *Sry* expression than Sry#49 gonads, in agreement with the previous analysis at 11.5 dpc (Fig. 2). Sry-L741 also showed no indication of a temporal pattern reminiscent of endogenous *Sry*, most notably because expression remained roughly consistent between 11.5 and 13.5 dpc.

### Non-gonadal transgene expression

**Figure 6:**
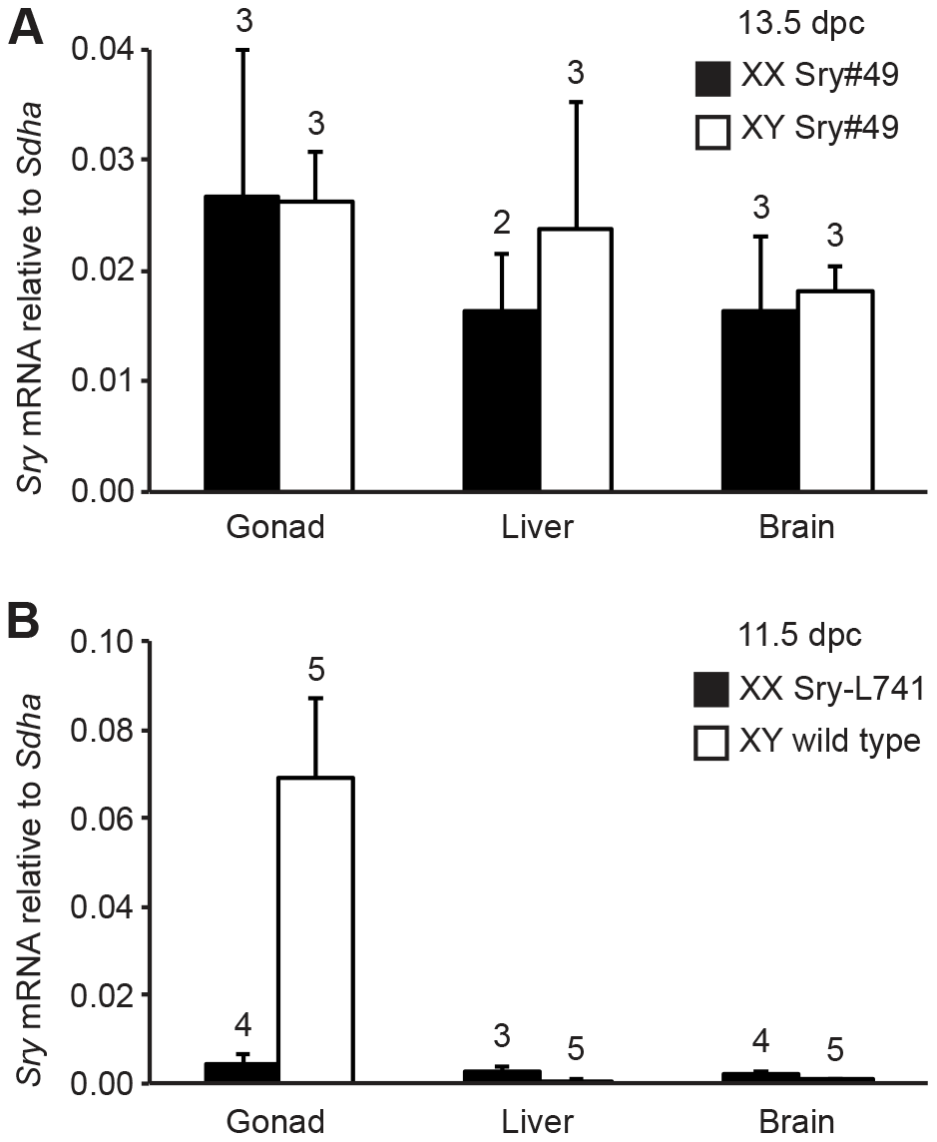
*Sry* transgene expression in gonadal *versus* non-gonadal tissue. Analyses were performed by qRT-PCR (2^−**Δ**Ct^, normalized against *Sdha*). (A) Expression of *Sry* mRNA in XX Sry#49 (black) and XY Sry#49 (white) tissues at 13.5 dpc. (B) Expression of *Sry* mRNA in XX Sry-L741 (black) and XY wild type (white) tissues at 11.5 dpc. Error bars are 95% confidence intervals. Numbers above bars indicate sample size.

In addition to the defined temporal window, endogenous *Sry* expression is also largely restricted during embryogenesis to the precursor Sertoli cells of the developing gonads. To determine if the *Sry* transgenes emulated the tissue-specificity of endogenous *Sry*, expression of Sry#49 mRNA was compared for gonad, liver and brain tissue from XX and XY embryos at 13.5 dpc (Fig. 6A). Expression did not differ significantly between the sexes, or between the gonad and liver, but was slightly higher for gonad compared with brain tissue (*P*<0.05). Expression of the Sry-L741 transgene was similarly compared for gonad, liver and brain tissue from XX embryos at 11.5 dpc (Fig. 6B; XY wild type *Sry* expression shown for comparison). Expression of the Sry-L741 transgene was very low in all tissue types, and did not differ significantly between tissues. For both transgenes examined, expression was clearly not restricted to gonad tissue, in contrast to endogenous *Sry*.

These data suggest that *Sry* transgenes from the *Col1a1* locus are transcribed in most, if not all cell types. If this is the case, then the level of *Sry* mRNA expression for XX Sry#49 gonads at 11.5 dpc (the most strongly-expressed transgene; Fig. 2A) may reflect the total amount for all gonad (and mesonephros) cells combined, such that the amount of *Sry* mRNA per precursor supporting cell is very low compared with the equivalent cells in the XY wild type gonad at 11.5 dpc. This situation likely explains why there is insufficient *Sry* expression per cell in Sry#49 gonads to upregulate *Sox9* and induce XX sex reversal. It would also be consistent with the failure to detect SRY protein in the XX Sry#49 gonads by IF staining: the amount of transgenic SRY protein per cell may be too low to be detectable.

## Discussion

More than two decades have passed since *SRY* was established as the testis-determining gene in humans and mice, but the specific *cis-*sequences required for the correct regulation of this pivotal sex-determining gene are still unidentified. In this study, we set out to delineate the 5′ sequences required for correct spatiotemporal regulation of *Sry* in the mouse by employing a site-specific, targeted transgenic strategy designed to avoid the limitations associated with conventional transgenesis. We created five transgenic lines bearing a single-copy of an *Sry* transgene, with sequentially deleted promoter regions ranging from 8 kb (the original L741 sequence) to only 0.3 kb of the region 5′ to the transcriptional start site. Unexpectedly, all five *Sry* transgenes failed to induce sex reversal in XX embryos. Our analyses indicated that the *Sry* transgenes were expressed in all cell types, but the level of expression in precursor supporting cells of the developing gonadal ridge was too low to upregulate *Sox9* expression and induce XX male development.

Our present findings contrast with those of our previous study, in which we found that a series of *Sry* transgenes, bearing sequentially deleted 5′ promoter regions ranging from 8 kb (L741) to only 57 bp (relative to the TSS), were all capable of inducing XX sex reversal when randomly integrated into the genome by pronuclear injection [36]. Moreover, in that study, there was no reduction in the frequency of sex reversal with sequentially shortened constructs. We assumed that any differences between the 5′ regions in their ability to facilitate transactivation of the *Sry* transgene were masked by the position effects typically associated with transgenesis by pronuclear injection [37]–[39]. Such effects arise because transgenes are randomly integrated at one or multiple unknown locations within the genome, and in variable copy number - sometimes up to several hundred copies arrayed in tandem. Both copy number and genomic location contribute to variable and unpredictable expression levels; this precluded any meaningful quantitative analysis of *Sry* expression from the deletion constructs. In contrast, the targeted transgenic strategy used in the current study was expected to produce consistent transgene expression levels, allowing us to accurately assay the activity and tissue-specificity of different promoter constructs *in vivo*. The lack of XX sex reversal for all five constructs analysed in the present study was unexpected; at the very least, we anticipated that the L741 transgene would induce sex reversal. We suggest two possible explanations for the discrepancy: (1) the *Col1a1* site was not suitable as a genomic location for examining transcriptional activation of *Sry*; or (2) the *cis-*sequences necessary for *Sry* regulation lie beyond the 8 kb of 5′ sequence in L741.

Regarding the first possibility, we adopted this gene targeting strategy based on a published report that demonstrated its effectiveness for an EGFP transgene [31]. The *Col1a1* locus was chosen for the original study because it had been shown to support high transgene expression even in cell types not normally expressing *Col1a1* [40]. In that investigation, the transgene was inserted into a targeting vector that additionally included a tetracycline-inducible promoter. In our laboratory, we have successfully used this tetracycline-inducible system to target and induce expression of a *Cyp26b1* transgene at the *Col1a1* integration site [41]. In the case of *Sry*, however, we used a targeting vector that lacked the tetracycline-inducible promoter because we aimed to identify important regulatory sequences by promoter deletion analysis. Despite this difference, there is no immediately apparent explanation why the *Col1a1* site might be unsuitable for proper transcription of the *Sry* transgenes. The fact that Sry#49 showed strong mRNA expression relative to the other transgenes, in both XX and XY embryos, suggests that the *Col1a1* integration site was accessible to transcriptional machinery to some degree; otherwise, all five transgenes would have been expressed at an equally low level.

If the integration site did not hinder *Sry* expression, an alternative explanation for our data is that the *cis-*sequences necessary for stage and tissue-specific activation of *Sry* to a level sufficient for *Sox9* upregulation were absent from all five transgenic constructs. If that were so, such sequences must lie beyond the 8 kb of 5′ sequence (or outside the entire 14.6 kb fragment) of L741. This assumes that expression of randomly integrated *Sry* transgenes in previous studies [1], [6], [11] was driven by the fortuitous presence of functional regulatory sequences at the integration sites. Consistent with this assumption, one study found that XX sex reversal could be induced by an *Sry* transgene in which the entire *Sry* 5′ region was replaced by the weak basal promoter of the *Hsp70.3* gene [42]. However, the conclusion that L741 lacks the essential *cis*-regulatory sequences is difficult to reconcile with previous transgenic and *in vitro* studies of *Sry* regulation.

Firstly, Albrecht and Eicher (2001) analysed an EGFP reporter transgene under the control of the L741 5′ promoter region (lacking only 0.5 kb of the 8 kb sequence at the 5′ end). *Sry^EGFP^* mRNA was expressed in mouse genital ridges at a low level at 10.5 dpc, increased at 11.5 dpc, and thereafter decreased by 15.5 dpc, and diminished further by postnatal days 1 and 28. EGFP protein was expressed specifically in gonadal somatic cells co-expressing WT1 and GATA4, appeared in the centre of the gonadal ridge at about 11.0 dpc, and spread to 70–80% of the ridge by 11.5 dpc. Thus the *Sry^EGFP^* transgene was expressed in the same cell lineage and with almost the same stage-specificity as endogenous *Sry*. It differed only in that the transgene was not totally transcriptionally silenced after 12.5 dpc, which the authors attributed to the lack of *Sry* 3′ flanking sequence in the transgene.

In a later study, Sekido et al. (2004) analysed two reporter variants of an L741 transgene, one bearing a *Myc* tag immediately 3′ to the *Sry* coding region and another in which the HMG and bridge domains were replaced by the human placental alkaline phosphatase gene. Expression of both *Sry^Myc^* and *Sry^hPLAP^* was gonad-specific and began after 10.5 dpc, peaked at 11.5 dpc, and ceased by 12.5 dpc (both constructs included *Sry* 3′ sequences, unlike *Sry^EGFP^*). Further, *Sry^hPLAP^* expression was shown to occur only in XY gonadal cells fated to become Sertoli cells.

Finally, in an *in vitro* LacZ reporter study, Ito et al. (2005) transfected Sry-Cre deletion constructs into primary cultures of gonad, brain and liver cells from CAG/loxP/CAT/loxP/LacZ transgenic mice. Constructs with *Sry* promoters ranging in length from 4.1 kb to 0.5 kb all induced LacZ expression in gonad cells from 11.5 dpc, but not in brain or liver cells, or gonad cells from 13.5 dpc. This tissue and stage-specificity disappeared for the shortest *Sry* promoter of 0.4 kb; LacZ expression occurred in all the cell types from 11.5 dpc and in gonad cells from 13.5 dpc.

Together, these studies suggest that the 14.6 kb L741 fragment contains the necessary *cis-*sequences for correct spatiotemporal regulation of *Sry*. The conspicuous difference between the previous L741 reports and our study, where L741 (and four shorter variants) did not emulate *Sry* expression, is that we integrated a single copy of each transgene into a defined autosomal locus. We conclude that the most likely explanation for our observations is that the *Col1a1* integration site was inappropriate for our purpose of testing *Sry* promoter deletion constructs. It remains to be determined why this integration site might have been unsuitable; a possible explanation is that the epigenetic conformation of this site is not accessible to transcription factors necessary for *Sry* regulation. Further studies may benefit from targeting different transgene integration sites.

Despite the lack of XX sex reversal or correct spatiotemporal *Sry* expression in any of the five transgenic lines, the strikingly higher *Sry* mRNA expression observed for XX Sry#49 gonads relative to the four other XX lines may still provide clues to the location of important 5′ regulatory sequences. This result suggests that: (a) the 1044 bp between the 5′ ends of Sry#49 and Sry#5 (−1313 to −269 relative to the TSS) may contain *cis-*sequences that strongly transactivate *Sry*; and (b) the 782 bp sequence between the 5′ ends of Sry#18 and Sry#49 (−2095 to −1313 relative to the TSS) may contain *cis-*sequences that have a repressive effect on *Sry* transcription. These possibilities require further investigation; rather than targeted integration of *Sry* transgenes, an alternative approach to elucidating *Sry* regulation might be to employ the newly emerged nuclease-based genome-editing technologies such as TALENS or CRISPR/Cas-mediated genome engineering to directly modify the 5′ and 3′ flanking sequences of *Sry* in its native Y chromosome location.
